# A Patient-Derived Organoid-Based Radiosensitivity Model for the Prediction of Radiation Responses in Patients with Rectal Cancer

**DOI:** 10.3390/cancers13153760

**Published:** 2021-07-27

**Authors:** Misun Park, Junhye Kwon, Joonseog Kong, Sun Mi Moon, Sangsik Cho, Ki Young Yang, Won Il Jang, Mi Sook Kim, Younjoo Kim, Ui Sup Shin

**Affiliations:** 1 Department of Radiological & Clinical Research, Korea Cancer Center Hospital, Korea Institute of Radiological and Medical Sciences, Seoul 01812, Korea; usre@kirams.re.kr (M.P.); jhkwon@kirams.re.kr (J.K.); 2 Department of Pathology, Korea Cancer Center Hospital, Korea Institute of Radiological and Medical Sciences, Seoul 01812, Korea; balltta9@kirams.re.kr; 3 Department of Surgery, Korea Cancer Center Hospital, Korea Institute of Radiological and Medical Sciences, Seoul 01812, Korea; msm@kirams.re.kr (S.M.M.); whtkdtlr@kirams.re.kr (S.C.); 4 Department of Internal Medicine, Korea Cancer Center Hospital, Korea Institute of Radiological and Medical Sciences, Seoul 01812, Korea; gooddryang@naver.com; 5 Department of Radiation Oncology, Korea Cancer Center Hospital, Korea Institute of Radiological and Medical Sciences, Seoul 01812, Korea; zzang11@kirams.re.kr (W.I.J.); mskim@kirams.re.kr (M.S.K.)

**Keywords:** machine learning, patient-derived tumor organoid, precision medicine, radiation response, rectal cancer

## Abstract

**Simple Summary:**

Predicting the tumor regression grade of locally advanced rectal cancer after neoadjuvant chemoradiation is important for customized treatment strategies; however, there are no reliable prediction tools. A novel preclinical model based on patient-derived tumor organoids has shown promising features of the recapitulation of real tumors and their treatment response. We conducted a small co-clinical trial to determine the correlation between the irradiation response of individual patient-derived rectal cancer organoids and the results of actual radiotherapy. Among the quantitative experimental data, the survival fraction was best matched and correlated with the patients’ real treatment outcome. In the machine learning-based prediction model for radiotherapy results using the survival fraction data, the prediction accuracy was excellent at more than 89%. Enhanced machine learning with the accumulation of further new experimental data would help in creating a more reliable prediction model, and this new preclinical model can lead to more advanced precision medicine.

**Abstract:**

Patient-derived tumor organoids closely resemble original patient tumors. We conducted this co-clinical trial with treatment-naive rectal cancer patients and matched patient-derived tumor organoids to determine whether a correlation exists between experimental results obtained after irradiation in patients and organoids. Between November 2017 and March 2020, we prospectively enrolled 33 patients who were diagnosed with mid-to-lower rectal adenocarcinoma based on endoscopic biopsy findings. We constructed a prediction model through a machine learning algorithm using clinical and experimental radioresponse data. Our data confirmed that patient-derived tumor organoids closely recapitulated original tumors, both pathophysiologically and genetically. Radiation responses in patients were positively correlated with those in patient-derived tumor organoids. Our machine learning-based prediction model showed excellent performance. In the prediction model for good responders trained using the random forest algorithm, the area under the curve, accuracy, and kappa value were 0.918, 81.5%, and 0.51, respectively. In the prediction model for poor responders, the area under the curve, accuracy, and kappa value were 0.971, 92.1%, and 0.75, respectively. Our patient-derived tumor organoid-based radiosensitivity model could lead to more advanced precision medicine for treating patients with rectal cancer.

## 1. Introduction

Since the German trial of 2004, neoadjuvant chemoradiation therapy (NCRT), followed by radical surgery with total mesorectal excision, has been a standard treatment for locally advanced rectal cancer without metastasis [1,2]. With NCRT, the rate of local recurrence is significantly reduced, and the survival rate of cancer patients is significantly increased among good radiation responders [3,4,5]. Tumor response is evaluated based on pathologic findings of tumor regression, or the amount of TNM downstaging in postoperative surgical specimens compared with the clinical TNM staging [6]. The downstaging rate is 60–80%, of which 15–20% show a pathological complete response. However, approximately 20–40% of patients do not benefit from NCRT.

Currently, even if a complete response is clinically observed after NCRT, radical resection is recommended, which can be accompanied by serious surgical morbidity or impaired quality of life. However, it has been suggested that radical surgery is unnecessary if NCRT eradicates all tumor cells. Beets et al. suggested the ‘wait and see’ approach for rectal cancer patients [7]. According to these authors, if rectal cancer patients have a clinical complete response, as determined based on strict preoperative endoscopic criteria, after NCRT, undertaking nonoperative management or delayed surgery does not compromise long-term oncologic results [8]. In contrast, to improve the radiation response rate, many studies have been conducted by adding more intensive drug therapies during the peri-radiation period. The single-agent 5-fluorouracil (5-FU) or its derivatives have been used as a radiosensitizer. However, more intensive chemotherapeutic drugs (oxaliplatin or irinotecan) or biologics (cetuximab, bevacizumab, or panitumumab) have been added to enhance the radiation response [9,10,11,12,13,14,15,16]. However, administering these intensive treatments to all patients with rectal cancer is not cost-effective and is associated with increased toxicity. Moreover, the issue of overtreatment cannot be avoided.

In terms of precision medicine, rectal cancer is an ideal candidate, as treatment strategies can be tailored according to the expected radioresponsiveness. If a pathological complete response is expected, patients could avoid radical surgery, or if the expected radioresponsiveness is poor, more intensive preoperative chemotherapy could be administered. Therefore, the development of reliable prediction tools for radioresponsiveness is important.

As a preclinical model for precision medicine, patient-derived tumor organoids (PDTOs) have shown advantages over patient-derived tumor xenograft models, but have many limitations in clinical usage owing to their high cost and time taken to establish individual patient-derived models [17,18]. For pancreatic cancer and metastatic gastrointestinal cancer, the PDTO models showed a high correlation with clinical outcomes in terms of drug response [19,20]. Regarding radiation response, Ganesh et al. [21] and Yao et al. [22] recently generated PDTOs from patients with rectal cancer, and reported that PDTOs mirrored individual radiotherapy outcomes. Their results suggest that PDTOs can be used to predict individual responses to chemoradiation. However, prior studies have not identified the method that can best determine the correlation between PDTO response and patient outcome.

In this co-clinical trial, we attempted to reproduce previous study results to determine whether there is a correlation between experimental results obtained after irradiation in PDTOs and actual individual NCRT results of patients. In addition, we constructed a simple machine learning model that predicts patients’ actual NCRT results based on the experimental data.

## 2. Materials and Methods

### 2.1. Patient Enrolment and Treatment

Between November 2017 and March 2020, we prospectively enrolled 33 patients diagnosed with mid-to-lower rectal adenocarcinoma pathologically confirmed by endoscopic biopsy. All patients underwent a staging workup using pelvic MRI; chest, abdominal, and pelvic computed tomography (CT); and 18-fluoro-2-deoxy-glucose positron emission tomography/CT. For patients diagnosed with locally advanced rectal cancer, NCRT was performed over a long course with a dose of 50.4 Gy in 28 fractions administered during weekdays. Chemotherapy was administered with a single-agent infusional 5-FU (425 mg per body square meter) for 5 days every 4 weeks before surgery. Radical surgeries were performed 6–8 weeks after completing radiotherapy with the aim of total mesorectal excision. Adjuvant chemotherapy was recommended for all medically fit patients after radical resection. For one patient who was diagnosed with a small resectable liver metastasis during staging workup, short-course radiotherapy with 25 Gy was administered in 5 Gy fractions over 5 days, followed by three cycles of neoadjuvant therapy: FOLFOX (5-FU, leucovorin, and oxaliplatin) with bevacizumab (the first cycle of FOLFOX only) every 2 weeks. Radical surgery, including liver metastasectomy, was performed 8 weeks after completing radiotherapy.

### 2.2. Pathologic Examination of Surgical Specimens

Standard pathologic tumor staging of the surgical specimen was performed and recorded according to the 8th edition of the TNM classification of the American Joint Committee on Cancer by dedicated gastrointestinal pathologists [23]. Pathologic response after NCRT was evaluated using the tumor regression grade (TRG) system suggested by the Gastrointestinal Pathology Study Group of the Korean Society of Pathologists [24]. The definitions of the TRG system are as follows: (A) TRG 0, complete response (no residual tumor cells were identified); (B) TRG 1, near complete response (only a few scattered tumor cells were present); (C) TRG 2, partial response (residual tumor glands with predominant fibrosis were easily identified); and (D) TRG 3, poor or no response (tumor cells did not demonstrate any response to chemoradiotherapy).

### 2.3. Tissue Acquisition

Pre-NCRT rectal cancer samples were obtained from enrolled patients at the endoscopic evaluation stage. Four or five rectal cancer biopsy samples were collected. A pathologist verified that the collected samples were histologically adenocarcinoma or normal crypts using hematoxylin and eosin (H&E) staining. The biopsy samples were pooled and immediately placed in cold phosphate-buffered saline with 50 µg/mL gentamicin (Gibco, Grand Island, NY, USA).

### 2.4. Organoid Cultures

Tumor organoids were isolated and cultured as previously described [25]. The composition of the PDTO culture medium is described in Supplementary Table S1. To prevent anoikis, 10 μM of Y-27632 was added to the culture medium for the first 2–3 days. When organoids were >200 µm, they were passaged by pipetting using Gentle Cell Dissociation Reagent (STEMCELL Technologies, Vancouver, BC, Canada) according to the manufacturer’s instructions. Most of PDTO used in experiments were cultured more than 14 days.

### 2.5. Immunocytochemistry and Immunohistochemistry

For immunocytochemistry, PDTOs were fixed in 4% paraformaldehyde at 25 °C for 24 h, embedded in paraffin, and then dissected into 3-µm-thick sections. After treatment with Smartblock solution (CANDOR Bioscience GmbH, Wangen im Allgäu, Germany) for 30 min at 25 °C, the slides were incubated with primary antibodies at 4 °C overnight and then incubated with secondary antibodies for 1 h at 25 °C. Images were acquired using the EVOS FL Cell Imaging System (Thermo Fisher Scientific, Carlsbad, CA, USA).

Immunohistochemistry was performed to characterize organoids and their tissues of origin with the colorectal markers caudal type homeobox 2 transcription factor, cytokeratin 7, and cytokeratin 20 on 3-μm-thick formalin-fixed paraffin-embedded tissue and organoid sections. Sections were incubated for 1 h at 37 °C with primary antibodies. Detection was performed using an Envision/Horseradish Peroxidase system (Dako; Agilent Technologies, Inc., Santa Clara, CA, USA) and counterstained with hematoxylin for 10 min at 25 °C. Finally, the sections were dehydrated through a graded series of alcohol, cleared in xylene, and mounted. Images were acquired using an IX73 inverted microscope (Olympus Corporation, Tokyo, Japan). The antibodies and dilutions used are described in Supplementary Table S2.

### 2.6. Survival Fraction Analysis

For survival fraction analysis, organoids were resuspended in TrypLE Express (Thermo Fisher Scientific, Carlsbad, CA, USA) via pipetting with a p200 pipette and incubated at 37 °C for 10 min. Cells were centrifuged at 600× *g* for 5 min, and the supernatant was discarded. The pellet was resuspended in Matrigel and distributed into a 48-well plate (500–1000 cells/20 μL of Matrigel per well). After the Matrigel had polymerized, 100 μL of culture medium was added. Over the following days, organoids were treated with 0 Gy, 2 Gy, 4 Gy, and 6 Gy using a ^137^Cs γ-ray source (Atomic Energy of Canada, Ltd., Renfrew County, ON, Canada) at a dose rate of 3.81 Gy/min. After 14 days, viable organoids were counted using Cell3 iMager Neo cc-3000 (Screen Holdings Co., Ltd., Kyoto, Japan). Analysis recipes were as follows: organoid diameter min 93, max 2907; organoid area min 6833, max 6,640,106; and circularity min 0.24, max 1. The plating efficiency was defined as the number of formed organoids/seeded cells × 100%. Survival fraction was calculated as follows: number of formed organoids/number of seeded cells in plate × (plating efficiency/100)]. A single-hit multitarget model was used to fit the survival curves, and D_0_, called the ‘mean lethal dose’, was the dose required to reduce the fraction of surviving organoids to 37% [26], calculated using GraphPad Prism software (version 8.0; GraphPad Software, Inc., La Jolla, CA, USA). For each PTDO, experimental replication of 4 wells was used. We obtained a total of 76 sets of survival fraction data.

### 2.7. Viability Assay

For the viability assay, organoids were resuspended in TrypLE. Cells were resuspended in Matrigel and distributed into a 96-well plate (5000 cells/10 μL of Matrigel per well). After the Matrigel had polymerized, 100 μL of culture medium was added. Over the following days, organoids were treated with 0 Gy, 2 Gy, 4 Gy, and 6 Gy. After 7 days, organoid viability was evaluated using CellTiter 96 AQUEOUS One Solution contains a tetrazolium compound [3-(4,5-dimethylthiazol-2-yl)-5-(3-carboxymethoxyphenyl)-2-(4-sulfophenyl)-2H-tetrazolium, inner salt; MTS] (Promega, Madison, WI, USA) according to the manufacturer’s instructions. Optical density was measured using a BioTek Eon microplate absorbance reader (BioTek Instruments Inc., Winooski, VT, USA) at 490 nm. Matrigel without organoids (10 µL) was used as a control.

### 2.8. Second Passage

For analysis at the second passage, organoids were treated with 5 Gy. After 72 h, organoids were passaged by pipetting using Gentle Cell Dissociation Reagent with a 1:2–1:4 split ratio. After 72 h, viable organoids were counted using the EVOS FL Cell Imaging System (Thermo Fisher Scientific, Carlsbad, CA, USA).

### 2.9. EdU Staining

Organoids were incubated with 10 µM EdU for 2 h and evaluated using Click-iT Plus EdU Imaging Kits (Thermo Fisher Scientific, Carlsbad, CA, USA) according to the manufacturer’s instructions. Images were acquired using the EVOS FL Cell Imaging System (Thermo Fisher Scientific, Carlsbad, CA, USA).

### 2.10. Western Blot Analysis

For Western blot analysis, organoids were washed with cold phosphate-buffered saline and lysed in radioimmunoprecipitation assay buffer (Thermo Fisher Scientific, Carlsbad, CA, USA). Proteins were quantified using the Bradford method, and 20–40 μg of protein was resolved using SDS-PAGE. The membranes were incubated with primary antibodies overnight at 4 °C, followed by incubation with a secondary antibody (Santa Cruz Biotechnology, Santa Cruz, CA, USA) for 1 h at 25 °C. Proteins were visualized using enhanced chemiluminescence (Thermo Fisher Scientific, Grand Island, NE, USA). Western blot images were analyzed using the Bio-Rad ChemiDoc (Bio-Rad, Richmond, CA, USA).

### 2.11. Targeted Next-Generation Sequencing Analysis

To analyze the mutational status of tissues and organoids, they were harvested using a cell recovery solution (Corning, Inc., Corning, NY, USA). DNA extraction and library construction were performed using the Gentra Puregene kit (Qiagen, Hilden, Germany) and SureSelect XT library prep kit (Agilent Technologies, Santa Clara, CA, USA). Deep targeted sequencing using Axen Cancer Panel 2 (170 cancer-related genes; Macrogen, Seoul, Korea) and the NextSeq 500 mid-output system platform (Illumina, San Diego, CA, USA) was conducted on 19 PDTOs. Libraries comprising 150-bp end reads were sequenced via high-throughput sequencing using synthesis technology to a depth coverage of approximately 2000×.

### 2.12. Statistical Analysis

Data obtained from a minimum of three independent experiments are expressed as mean ± standard deviation. Unpaired two-tailed Student’s *t*-tests were used to determine significant differences between the two groups. One-way analysis of variance followed by Tukey’s and Bonferroni tests was performed to compare the means between multiple groups, and *p* values < 0.05 were considered significant. Statistical and receiver operating characteristic (ROC) curve analyses were performed using R 4.0.2 (https://www.r-project.org; accessed on 15 May 2020). Analysis of the mutation-annotated files was conducted using the R package ‘maftools’ (version 3.12), which included the generation of figure oncoplots [27]. Comparison of linear-quadratic (LQ) cell survival curves was performed using analysis of variance calculated with the R package ‘CFAssay’ (version 1.22.0) [28].

### 2.13. Development of Predictive Models Using Machine Learning

To build the prediction model for TRG, we used survival fraction data. A total of 76 experimental data points were randomly split in a 1:1 ratio into training and testing datasets. The machine learning model was built using the training data and subsequently tested on the remaining 50% of the data comprising the testing set. The supervised machine learning classification algorithm performed binary logistic regression and random forest classification with the R package, ‘randomForest’ version 4.6-14. For model training with a random forest method, we used 200 trees and two variables as training hyperparameters. We calculated the area under the ROC curve (AUC), accuracy, and kappa value of the testing dataset to evaluate the model performance.

## 3. Results

### 3.1. Patient Characteristics and Treatment Outcomes

**Figure 1:**
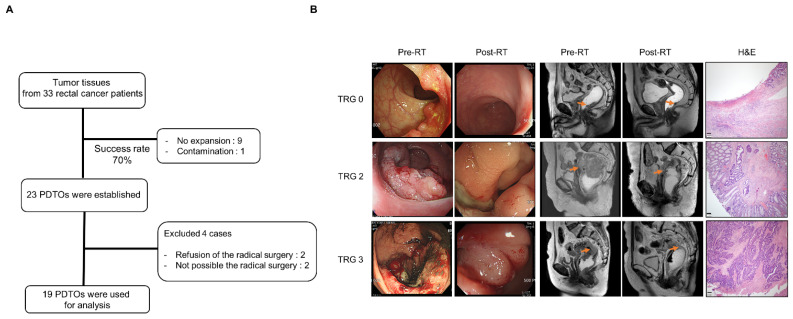
Patient characteristics and treatment outcomes. (**A**) Flow chart indicating the number of patients with rectal cancer, including reasons for non-evaluability, and the success rate of establishing cultures from patients. (**B**) Pre- and post-RT endoscopic clinical responses, magnetic resonance images. and H&E staining images are shown for TRG 0, TRG 2, and TRG 3 patients. Magnification, ×4. Scale bars, 200 µm Abbreviations: H&E, hematoxylin and eosin; PDTO, patient-derived tumor organoid; RT, radiotherapy; and TRG, tumor regression grade.

Tumor tissues were collected by endoscopic biopsy from 33 patients with rectal cancer. Among 33 tumor tissues, 10 PDTOs could not be established due to bacterial contamination in one case and no expansion in the culture medium in nine cases (70% success rate). In addition, two patients were excluded as they were diagnosed with unresectable metastatic rectal cancer; radical surgeries were not planned for these patients, and it would not have been possible to evaluate their TRG. Two patients refused radical surgeries and were also excluded. Finally, 19 patients and their PDTOs were analyzed in this study (Figure 1A). Representative images of the 19 PDTOs are displayed in Supplementary Figure S1. Individual patient characteristics and clinical treatment results are summarized in Table 1. The median age of patients was 59 (interquartile range, 53.0–70.5) years. The male-to-female ratio was 14:5. Eighteen patients had stage III disease, and one patient had stage IV disease with resectable liver metastasis. After R0 surgery following NCRT, TRGs were as follows: five patients achieved TRG 0 (26.3%), and one patient had TRG 1. Three patients had TRG 3, and the other 11 patients had TRG 2 (Figure 1B). During a median of 19.0 (interquartile range, 12.5–26.5) months of follow-up, six patients developed tumor recurrence (five distant, one local), and one patient died due to recurrence (Table 1).

### 3.2. Histological and Genomic Characterization of PDTOs

**Figure 2:**
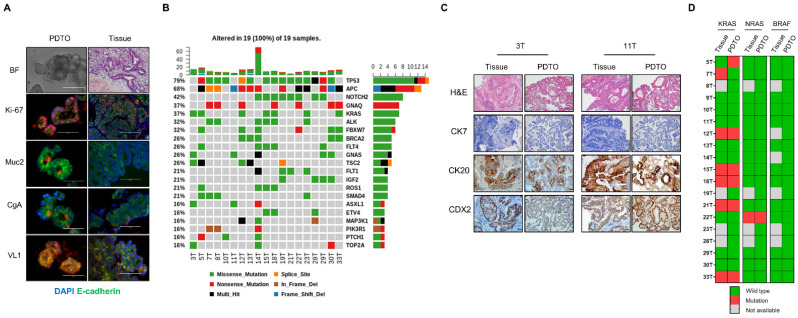
Histological and genomic characterization of PDTOs. (**A**) Fluorescence microscopy images of FFPE sections of organoids and corresponding tissues for goblet cells (mucin 2 [Muc2]+, red), entero-endocrine cells (CgA+, red), enterocytes (villin 1 [VL1]+, red), and proliferating cells (Ki-67, red). Counterstain, DAPI (blue) and epithelial, E-cadherin (green). Scale bars, 100 µm. (**B**) The mutation landscape of 19 PDTOs. The frequency of alterations in PDTO is noted with the type of genetic alteration (indicated by color code). The top 21 mutated genes observed in PDTOs, including the most known significant cancer driver genes, are shown. (**C**) Immunohistochemical profile of FFPE sections of organoids and corresponding tissues for cytokeratin 7, cytokeratin 20, and caudal type homeobox 2 transcription factor along with corresponding H&E staining. Magnification, ×40. Scale bars, 50 µm. (**D**) *KRAS*, *NRAS,* and *BRAF* mutation status of PDTOs and paired tumor tissues. Abbreviations: CDX, caudal type homeobox; CK, cytokeratin; DAPI, 4′,6-diamidino-2-phenylindole; FFPE, formalin fixed paraffin-embedded; H&E, hematoxylin and eosin; and PDTO, patient-derived tumor organoid.

To verify PDTOs, immunostaining was performed using paraffin-embedded organoid sections and tissues. Our PDTOs differentiated into enterocytes (villin 1), goblet cells (mucin 2), and enterochromaffin cells (chromogranin A) and contained amplifying cells (Ki-67; Figure 2A). To analyze the mutational status of the 19 PDTOs, we performed targeted next-generation sequencing analysis using Axen Cancer Panel 2. Variants were filtered based on a multivariate alteration detection of <2%, type of alteration (multi-hit, missense, nonsense, splicing site, in-frame del, and frame-shift), fusion gene, copy number alterations, and functional consequence (pathogenic, likely pathogenic, benign, and likely benign). Genes of the WNT signaling pathway (*APC* and *FBXW7*) were mutated in 68.4% (13/19) of all PDTOs. *APC* and *FBXW7* mutations were identified in 13 of 19 PDTOs (68.4%) and 6 of 19 PDTOs (31.5%), respectively (Figure 2B). All mutation alterations are displayed in Supplementary Figure S2. We performed H&E staining and immunostaining of the proteins cytokeratin 7, cytokeratin 20, and caudal type homeobox 2 transcription factor to confirm that our PDTOs originated from rectal cancer tissue and not from normal rectal mucosa. Our PDTOs showed similar histological morphologies and CK protein expression patterns to those of original tumor tissues (Figure 2C). Overall, these data demonstrated that PDTOs recapitulated the histological morphologies and marker expression of the paired patient tissues, as previously reported [21,29]. To define the capacity of colorectal cancer organoids to mirror the genome heterogeneity of the corresponding patient tumor, we compared the mutational status of three genes (*KRAS*, *NRAS,* and *BRAF)* in 19 PDTOs and corresponding tumor tissues. *KRAS*, *NRAS,* and *BRAF* mutations in PDTOs were matched to 86.6%, 100%, and 100% of those in corresponding tumor tissues, respectively (Figure 2D).

### 3.3. PDTOs Response to Irradiation

**Figure 3:**
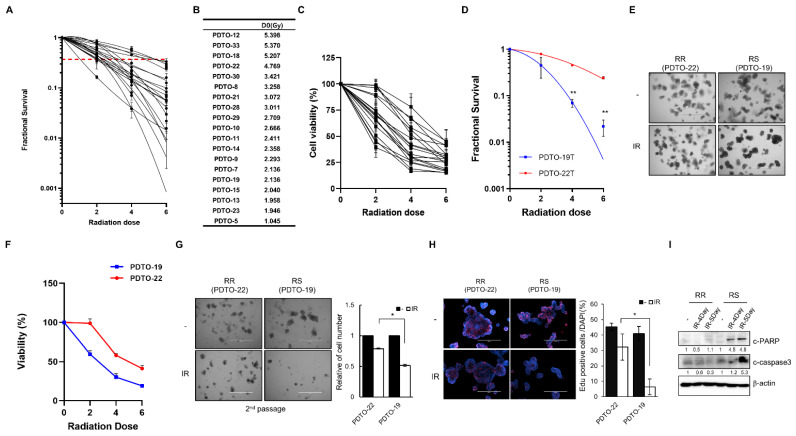
The response of PDTOs to radiation. (**A**) Dose–response of survival fraction in 19 PDTOs (*n* = 4, independent experiments for each PDTO) is shown. Data are presented as mean ± standard deviation. The red line represents a survival fraction of 0.37. (**B**) D_0_ values were calculated according to the multitarget single-hit model. (**C**) MTS cell viability assay of 19 PDTOs after 0 Gy, 2 Gy, 4 Gy, and 6 Gy irradiation (*n* = 6, independent experiments for each PDTO). Data are normalized to those of control cells. Data are presented as mean ± standard deviation. (**D**) Dose–response of survival fraction in PDTO-19 and PDTO-22 (*n* = 4 independent experiments for each PDTO) is shown. ** *p* < 0.01. (**E**) Morphology of PDTO-19 and PDTO-22 after irradiation with 5 Gy after 5 days. Scale bars, 1000 µm. (**F**) MTS cell viability assay of PDTO-19 and PDTO-22 after treatment with 0 Gy, 2 Gy, 4 Gy, and 6 Gy. Data are normalized to those of the control cells and presented as mean ± standard deviation. ** *p* < 0.01. (**G**) (**left**) Image of organoids after the second passage. Scale bars, 1000 µm. (**right**) The relative organoid number after the second passage. Data are presented as mean ± standard deviation. * *p* < 0.05. (**H**) (**left**) Fluorescence microscopy images of EdU incorporation in PDTO-19 and PDTO-22 after irradiation. Scale bars, 400 µm. Blue, DAPI; red, EdU. (**right**) Statistical analysis representing EdU-positive cells per DAPI-stained cell (*n* = 3). * *p* < 0.05. (**I**) Expression levels of c-PARP and c-caspase-3 in PDTOs. β-actin was the loading control. Abbreviations: c-PARP, cleaved poly-ADP-ribose polymerase; DAPI, 4′,6-diamidino-2-phenylindole; IR, ionizing radiation; PDTO, patient-derived tumor organoid; RR, radioresistant; and RS, radiosensitive.

To validate the response of PDTOs to irradiation in vitro, we performed a radiation dose-dependent (0 Gy, 2 Gy, 4 Gy, and 6 Gy) survival analysis of 19 PDTOs. Supplementary Figure S3 displays representative images of irradiated organoids, and we counted the number of viable organoids after irradiation to measure the survival fraction (Figure 3A and Supplementary Figure S4). We analyzed the D_0_ value (the dose required to reduce the fraction of surviving organoids to 37%); a higher D_0_ value indicates greater radioresistance [26]. Therefore, we defined radioresistant PDTOs and radiosensitive PDTOs according to the D_0_ value (Figure 3B). These survival fraction data were validated by direct comparison using the MTS cell viability assay (Figure 3C and Supplementary Figure S5). The results demonstrated the heterogeneity of the radioresponse in 19 PDTOs. According to our data, PDTO-22 and PTDO-19 showed radioresistant and radiosensitive characteristics, respectively (Figure 3D,E). To confirm these different radioresponses, we tested this result using several in vitro analyses. The organoid viability of PDTO-19 cells was significantly reduced compared with that of PDTO-22 at 2 Gy, 4 Gy, and 6 Gy (*p* < 0.0001; Figure 3F). To directly assess the regenerative ability of organoids, we counted organoids at the second passage after splitting the irradiated organoids. Seventy-two hours after splitting, the relative number of PDTO-19 organoids was significantly lower than that of PDTO-22 after irradiation (*p* = 0.034; Figure 3G). To determine whether the change in cell viability was accompanied by cell proliferation, we performed EdU staining in PTDOs after irradiation and showed that 13% of the cells in the S phase decreased after irradiation in PDTO-22. In contrast, 30% of S phase cells were reduced after irradiation in PDTO-19 (*p* = 0.029; Figure 3H). To evaluate the apoptotic cellular response to radiation, apoptosis-related protein levels were analyzed. Cleaved-PARP and -caspase-3 levels, which are considered hallmarks of apoptosis, were increased in PDTO-19 after irradiation compared to those in PDTO-22 (Figure 3I).

### 3.4. Correlation of Experimental Data with Actual TRG Outcomes

**Figure 4:**
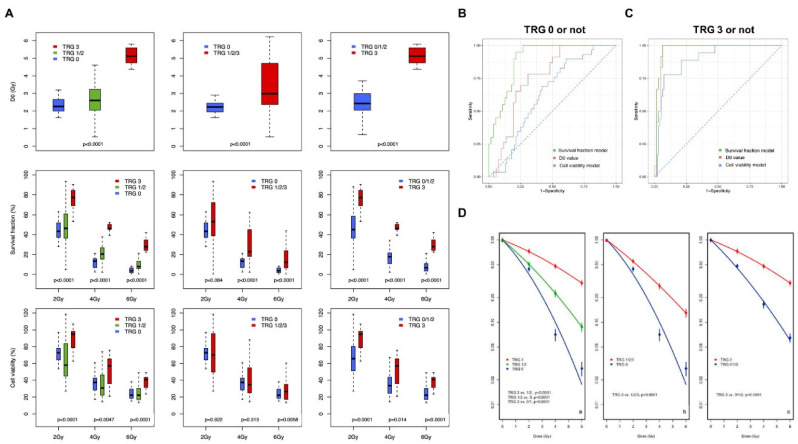
Correlation of experimental data with actual TRG outcomes following patients’ treatments. (**A**) Comparison of the mean and standard deviation of D0, survival fraction, and cell viability data according to the TRG groups. (**B**) Receiver operating curve analysis for predicting TRG 0 or not by D0, survival fraction, and cell viability data. (**C**) Receiver op-erating curve analysis for predicting TRG 3 vs. other using D0, survival fraction, and cell viability data. (**D**) Compari-son of reconstructed linear-quadratic cell survival curves according to the TRG groups (**a**), TRG 0 or not (**b**) and TRG 3 or not (**c**). Abbreviation: TRG, tumor regression grade.

To compare the experimental results of the survival fraction, D_0_ value, and cell viability, we regrouped TRGs into three categories: TRG 0, TRG 1/2, and TRG 3 (Figure 1B). The results of comparisons according to the three TRG groups and according to whether TRGs were at their two extreme categories, good responders (TRG 0 or not) and poor responders (TRG 3 or not), are shown in Figure 4A. Generally, *p* values obtained by comparing the mean (SD) values among the three TRG groups were more significant in the survival fraction and D_0_ data than in cell viability. Furthermore, comparing after actual TRGs were regrouped according to whether TRGs were in the two extreme categories or not, the *p* values were more significant for comparisons of survival fraction and D_0_ data (Figure 4A). Next, we performed ROC analyses to determine whether our experimental data could classify TRGs and which experimental data would be more appropriate to use for classifying TRGs. While D_0_ data had a single value, D_0_ only, the survival fraction data and cell viability data had multiple values at each radiation dose (2 Gy, 4 Gy, and 6 Gy). Therefore, we used a multiple logistic regression model to analyze survival fraction and cell viability data in this ROC analysis. In the ROC analysis of good responders (TRG 0), AUCs matched to D_0_, survival fraction, and cell viability tests were 0.753 (95% confidence interval (CI), 0.644–0.863), 0.897 (95% CI, 0.83–0.965), and 0.631 (95% CI, 0.525–0.737), respectively (Figure 4B). When analyzing poor responders (TRG 3), the AUCs of the respective experimental data were as follows: D_0_, 0.966 (95% CI, 0.926–1); the survival fraction model, 0.974 (95% CI, 0.941–1); and the cell viability model, 0.898 (95% CI, 0.827–0.968; Figure 4C). Sensitivity, specificity, positive predictive value, and negative predictive value were highest in the survival fraction model (Supplementary Table S3). We reconstructed the LQ curve according to the regrouped TRG using the 19 individual PDTO survival fraction curves (Figure 3A). When comparing the curves of the three groups, the LQ curves were clearly divided with statistical significance (*p* < 0.0001). In addition, TRG 0 or not (*p* < 0.0001) and TRG 3 or not (*p* < 0.0001) of the LQ curve were still significantly divided with respect to the TRG groups (Figure 4D).

### 3.5. Machine Learning-Assisted Prediction Model

**Figure 5:**
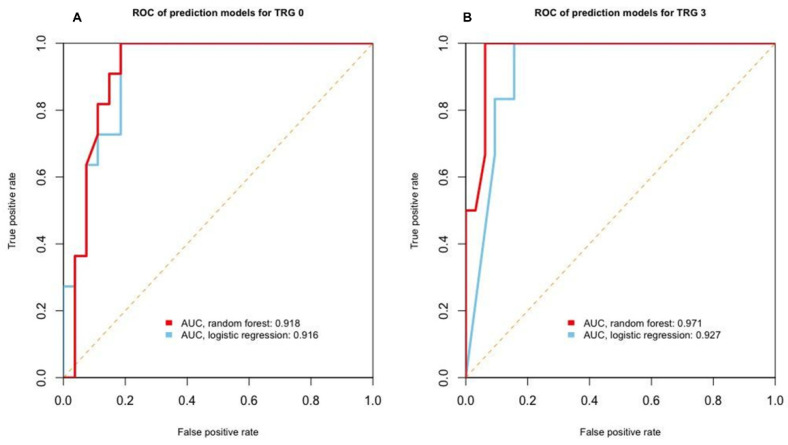
Machine learning-assisted prediction model. (**A**,**B**) Performance of machine learning-based prediction models for TRG 0 (**A**) and TRG 3 (**B**) on the testing dataset presented using receiver operating characteristic curves and their area under the curves. Red line: prediction model trained using the random forest algorithm, blue line: prediction model trained using binary logistic regression. Abbreviations: AUC, area under the ROC curve; ROC, receiver operating characteristic; and TRG, tumor regression grade.

As shown in Figure 4, the AUC, sensitivity, specificity, positive predictive value, and negative predictive value of the survival fraction model were highest among the values from the three experimental datasets. Therefore, we developed machine learning-based classification models using the survival fraction data. After building a prediction model using a training dataset, we evaluated the model performance using the testing dataset. In the prediction model for good responders (TRG 0) trained using logistic regression, the AUC was 0.916 (Figure 5A), the accuracy was 78.9%, and the kappa value was 0.38. The AUC, accuracy, and kappa value of the model trained using the random forest were 0.918, 81.5%, and 0.51, respectively. In the prediction model for poor responders (TRG 3) trained using logistic regression, the AUC, accuracy, and kappa value were 0.927, 89.5%, and 0.65, respectively (Figure 5B); those of the model trained using the random forest were 0.971, 92.1%, and 0.75, respectively.

## 4. Discussion

In this co-clinical trial, we reproduced the results of previous studies [21,22]. The histology, genetic features, and irradiation response of PDTOs mirrored real treatment outcomes of original tumors and patients. Furthermore, our quantitative experimental data correlated well with actual TRG results. With these results, we built a machine learning-based prediction model by inputting the survival fraction values of PTDOs. At the beginning of this study, we did not know which experimental indicator would best match the patient’s actual TRG results; thus, we conducted various experiments regarding organoid irradiation responses. Among them, we selected D_0_, survival fraction, and cell viability data, which were easily measurable and reproducible by repeated tests. We found that survival fraction data were the best-matched experimental results to the patient’s TRG results in statistical analyses. The machine learning-based prediction model using the survival fraction data showed an excellent performance.

Organoid technology has been a highlight for cancer research due to the close resemblance of organoids to original tumors [29,30,31,32,33,34,35]. Due to its rapid establishment with a high success rate, the organoid model is noted as a pre- or co-clinical model for precision medicine. Although not commented on in this study, the growth rate of PDTOs was heterogeneous, but could acquire enough volume to assess the irradiation response within 1–2 weeks in most cases. It added a testing time of approximately 4 weeks to obtain irradiation response data that can predict the TRG results of real patients. It is a clinically significant period to produce treatment recommendations, as stated by Yao et al. [22].

This study has some limitations. First, the study sample size was small. The goal of a machine learning model is to generalize patterns using training data to correctly predict new data that have never been presented to the model. Overfitting occurs when a model adjusts excessively to the training data, sees patterns that do not exist, and consequently performs poorly in predicting new data. The fewer samples for training, the more models that can fit. Our treatment-naive sample number was not smaller than that of previous studies [21,22]; however, it was not sufficient to obtain a reproducible prediction model, although we used the random forest method for model training and obtained acceptable model performance results. Random forest is an ensemble machine learning model that increases the model performance, but is not a solution for small sample size issues. To develop a reliable predictive model using organoids, a reliable volume of training samples is required [36]. Given that it is difficult to generate sufficient data in a single laboratory, it is necessary to collect and share data produced under consented standard experimental conditions among clinical organoid researchers.

Second, in the current organoid model itself, one can only observe the irradiation response of cancer cells themselves. For the actual therapeutic response of tumor cells to radiation, the role of the microenvironment is very important. Although organoid cultures provide more favorable conditions than traditional cell line models for tissue physiology and structure, which are close to in vivo situations, the model does not robustly retain the complexity and diversity of the tumor microenvironment (T-ME). The T-ME has been gradually recognized as a key contributor to cancer progression and a determinant of treatment outcomes [37,38]. In radiotherapy, vascular, stromal, and immunological changes in T-ME induced by radiation promote radioresistance and tumor recurrence; furthermore, radiotherapy has recently been proposed to target the T-ME to overcome radioresistance [39]. However, organoid cultures typically contain epithelium. Thus, to overcome these limitations, models for co-culture of tumor organoids and T-ME have recently been introduced. Öhlund et al. developed a co-culture system of pancreatic cancer organoids and cancer-associated fibroblasts that can recapitulate some of the features observed in patients [40]. Dijkstra et al. described a patient-personalized in vitro model that enabled the induction and analysis of tumor-specific T-cell responses using colorectal cancer organoids and T lymphocytes isolated from patients’ peripheral blood [41]. In addition, a unique co-culture method based on an air-liquid interface system permitted the propagation of PDO and tumor-infiltrating lymphocytes [42]. Organoid culture methods that partially retain the patient’s T-ME might overcome the hurdles of organoid culture and offer reliable results.

Finally, in this study, we only evaluated the response against irradiation. In a real situation, various chemotherapeutic agents are combined to obtain improved NCRT results [9,10,11,12,13,14]. However, we did not perform a drug sensitivity test as our study population comprised a homogenous patient group that used a single agent, 5-FU, as a concurrent treatment for all patients except one, and the difference in radioresponse affected by the combination of various drugs could not be observed. Based on this study result of radiosensitivity, and through further validations, we believe that we will be able to identify which element or combinations of current multimodal treatments would be most helpful and identify a more advanced tailored treatment via current ex vivo tests with PDTOs.

## 5. Conclusions

As revealed by previous studies, individual PDTOs recapitulated responses of original tumors to irradiation. The radiation response of PDTOs could predict the patient’s TRG with statistical significance. The PDTO-based radiosensitivity model could be a reliable diagnostic tool for the tailored treatment of rectal cancer.

## Figures and Tables

**Table 1:** Individual patient characteristics and treatment results.

Sample No. (PDTO)	5	7	8	9	10	11	12	13	14	15	18	19	21	22	23	28	29	30	33
**Sex/Age**	F/84	F/64	M/47	M/70	M/49	M/54	M/77	M/57	M/49	M/64	M/71	F/62	M/82	F/76	M/52	M/54	F/59	M/58	M/52
**BMI (kg/m^2^)**	24.8	24.4	16.2	18.9	25.5	22.2	16.5	25.7	17.7	24.6	22.7	17.3	20.9	24.3	19.7	26.1	25.3	20.8	21.2
**Diabetes**	No	No	No	No	No	No	No	No	Yes	No	No	No	No	No	No	No	Yes	No	No
**Clinical Stage**	T3N1	T3N2	T3N2	T3N1	T3N2	T3N1	T3N1	T3N1	T3N2	T2N1	T3N1	T3N1	T4N2	T3N2	T2N1	T3N1	T3N1	T3N1	T2N0M1
**Pre-RT-Tumor Size(cm) MRI**	6.5	4.5	4	3.8	8	4	4.2	5	6.5	3.8	5.4	3.5	7.5	6.5	6	3.5	5.3	5.6	2.6
**Post-RT-Tumor Size(cm) MRI**	3.5	3	2.8	3	7	2	2.3	2.6	4	1.6	5.3	2.5	5	2.5	2.5	2.5	2.8	3.5	1.1
**Post-RT-Tumor Size (cm) Surgical Specimen**	1.2	3.7	2.4	2.3	3.4	2.5	2.5	0.8	3.8	1	7.5	1.2	3.5	3.5	2.6	0.9	2.5	0.5	2.2
**TRG**	2	2	2	2	2	2	3(2)	1	0	0	3	0	2	3	0	0	2	2	2
**yp Stage**	T3N0	T3N2	T2N2	T2N0	T2N2	T3N1	T3N1	T2N1	T0N0	T0N0	T3N1	T0N0	T3N0	T3N0	T0N0	T0N0	T3N0	T2N0	T2N0M1
**Site of Recurrence**		Distant lymph node			Liver	Lung, distant lymph node	Lung				Local		Lung						
**Recurrence-free Survival (Months)**	26	25	28	28	15	13	13	26	24	23	10	17	10	12	14	9	6	5	5
**Dead**	No	No	No	No	No	No	Yes	No	No	No	No	No	No	No	No	No	No	No	No
**Overall Survival (Months)**	26	28	28	28	27	28	19	26	24	23	14	17	13	12	14	9	6	5	5

BMI, body mass index; RT, radiotherapy; MRI, magnetic resonance imaging; TRG0, (complete response) TRG1, (Nearly complete) TRG2, (Moderate) TRG3, (Minimal).

## Data Availability

All data in this study are included as Supplementary Tables and Figures. Any additional information is available upon request.

## Supplementary Materials

The following are available online at https://www.mdpi.com/article/10.3390/cancers13153760/s1, Figure S1: Observed morphologies of 19 PDTOs. Figure S2: The mutation of 19 PDTOs for all alterations is displayed. Figure S3: Morphologies of PDTOs after irradiation at 2 Gy, 4 Gy, and 6 Gy. Figure S4: Dose–response of survival fraction in 19 PDTOs. Figure S5: Dose–response of cell viability in 19 PDTOs. Figure S6: Whole blot showing all the bands with molecular weight marker. Table S1: List of chemical and reagents used for studies. Table S2. List of antibodies used for studies. Table S3: Results of ROC about two extreme categories.
