# Supplementary material for: A Patient-Derived Organoid-Based Radiosensitivity Model for the Prediction of Radiation Responses in Patients with Rectal Cancer
Source: Cancers (Basel). 2021 Jul 27;13(15):3760. doi: 10.3390/cancers13153760 (PMC8345202; doi:10.3390/cancers13153760)
Supplement: Supplementary file 1 [file cancers-13-03760-s001.zip › cancers-1287154-supplementary.pdf]

cancers

MDPI

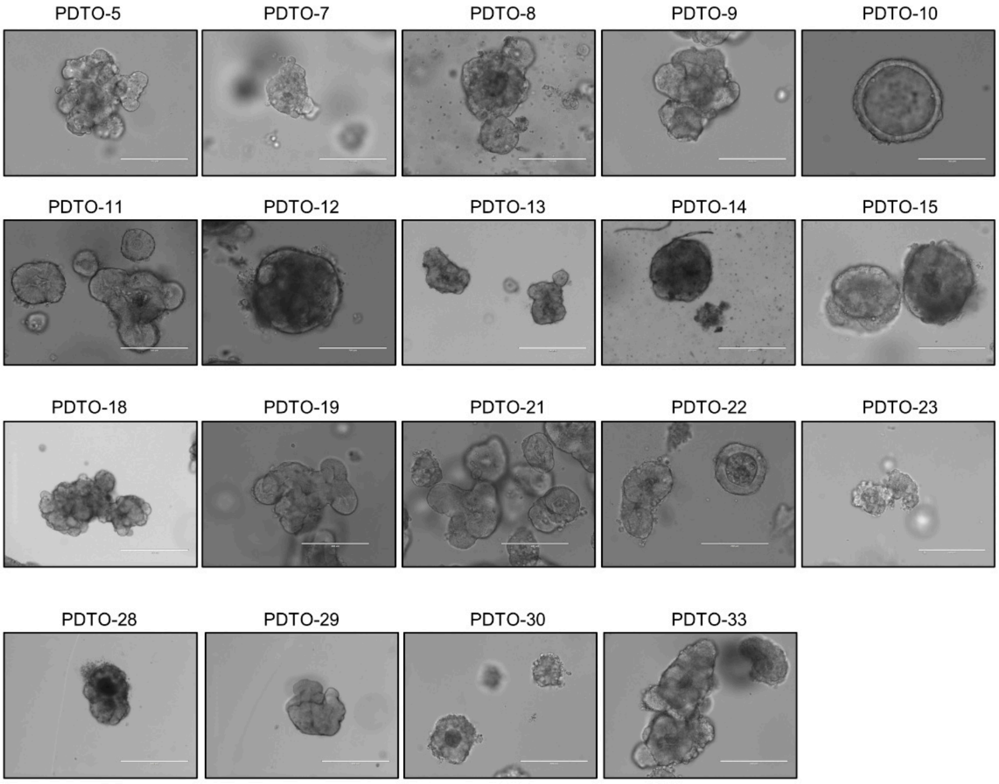

Figure S1. Observed morphologies of 19 PDTOs. PDTO, patient-derived tumour organoid.

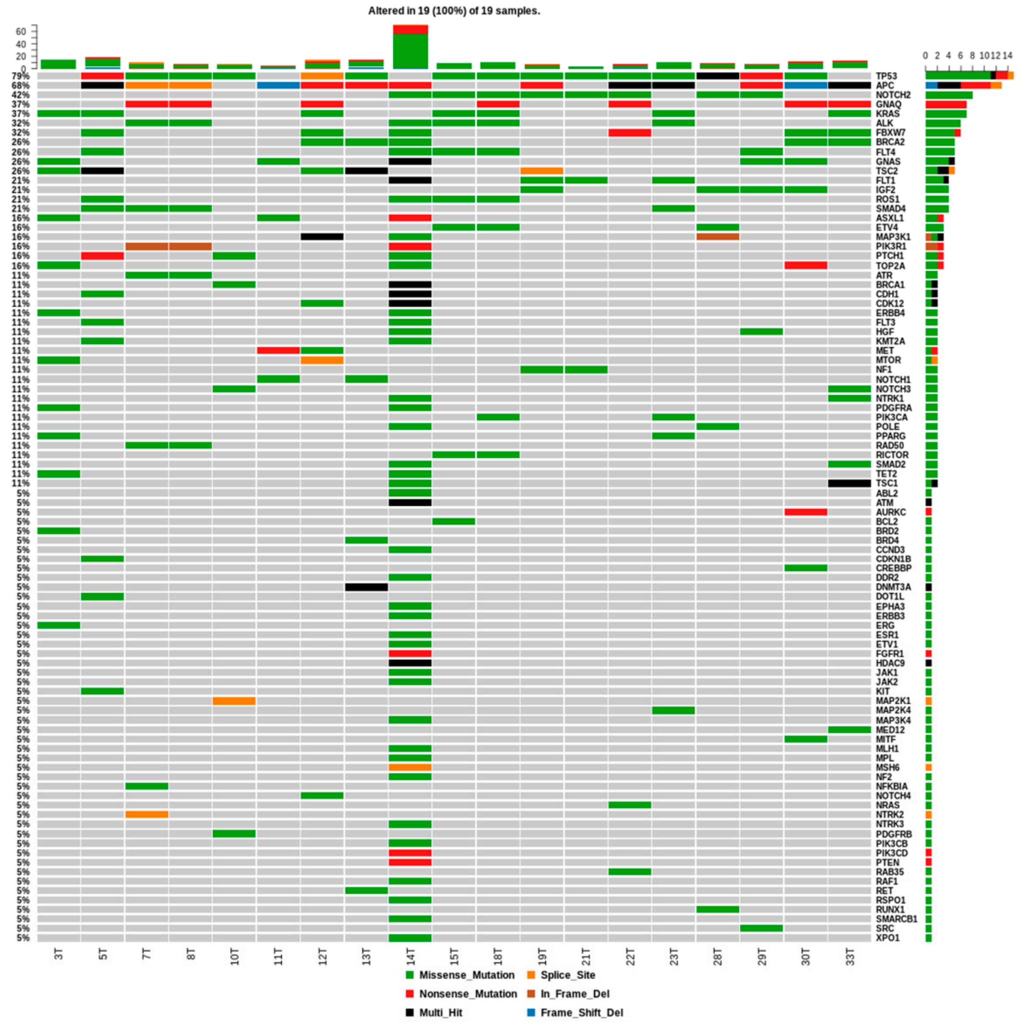

Figure S2. The mutation of 19 PDTOs for all alterations is displayed. The frequency of alteration is noted along with the type of genetic alteration relative to frame-shift, in-frame, missense, nonsense, or splice-site alterations (as indicated by the colour code). PDTO, patient-derived tumour organoid.

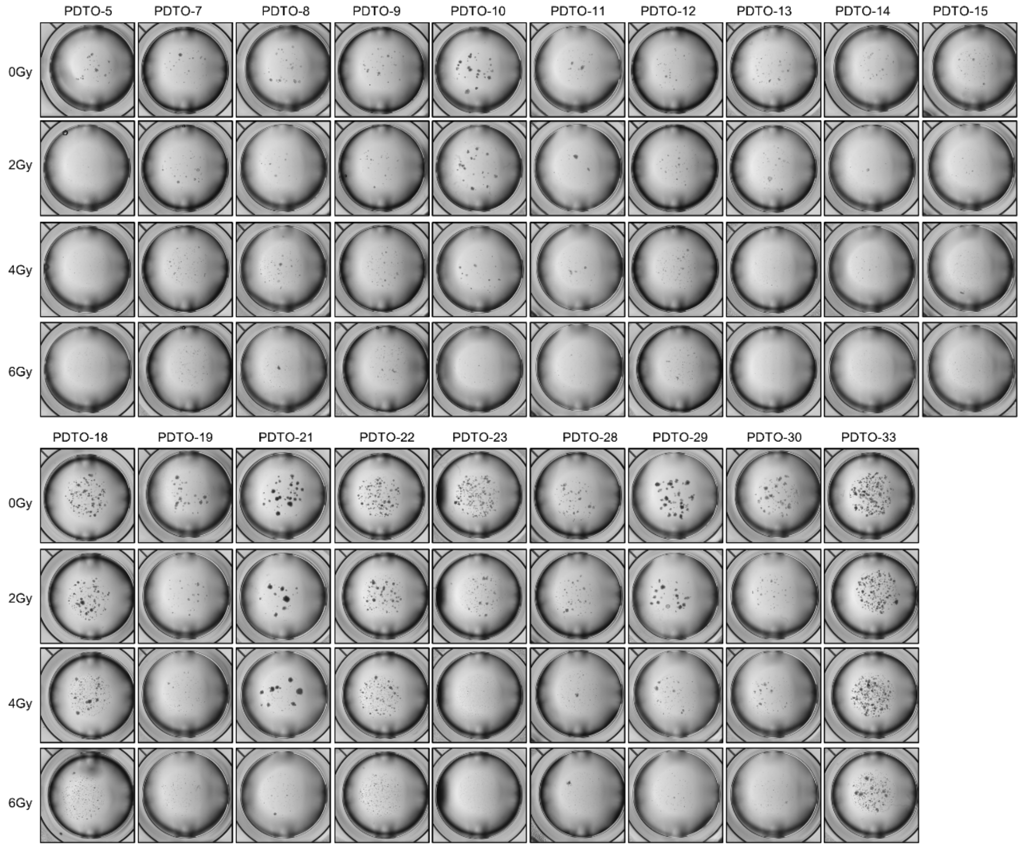

Figure S3. Morphologies of PDTOs after irradiation at 2 Gy, 4 Gy, and 6 Gy. PDTO, patient-derived tumour organoid.

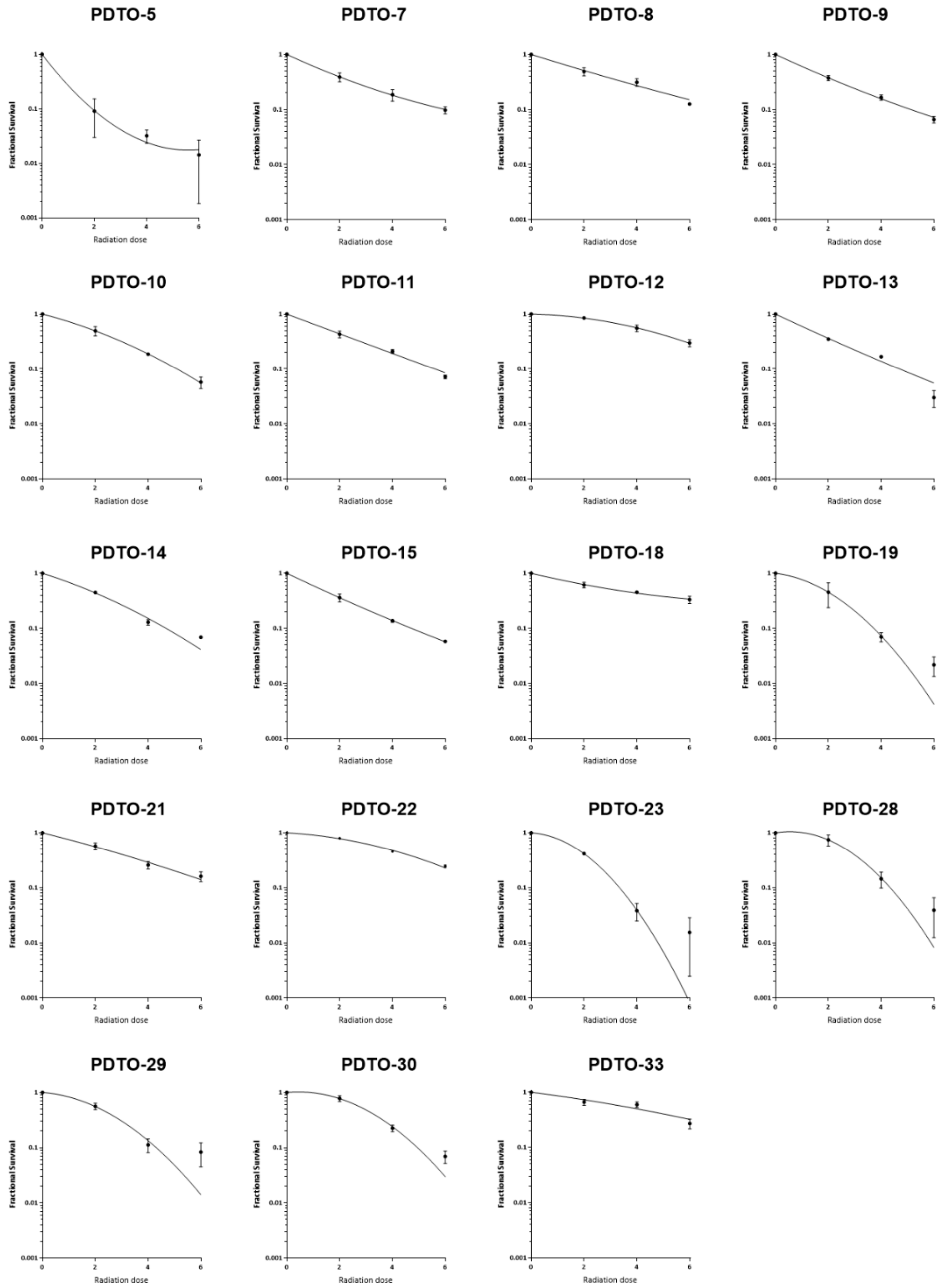

Figure S4. Dose-response of survival fraction in 19 PDTOs (n = 4, independent experiments for each PDTO) is shown at 0 Gy, 2 Gy, 4 Gy, and 6 Gy. Data are presented as mean ± standard deviation. PDTO, patient-derived tumour organoid.

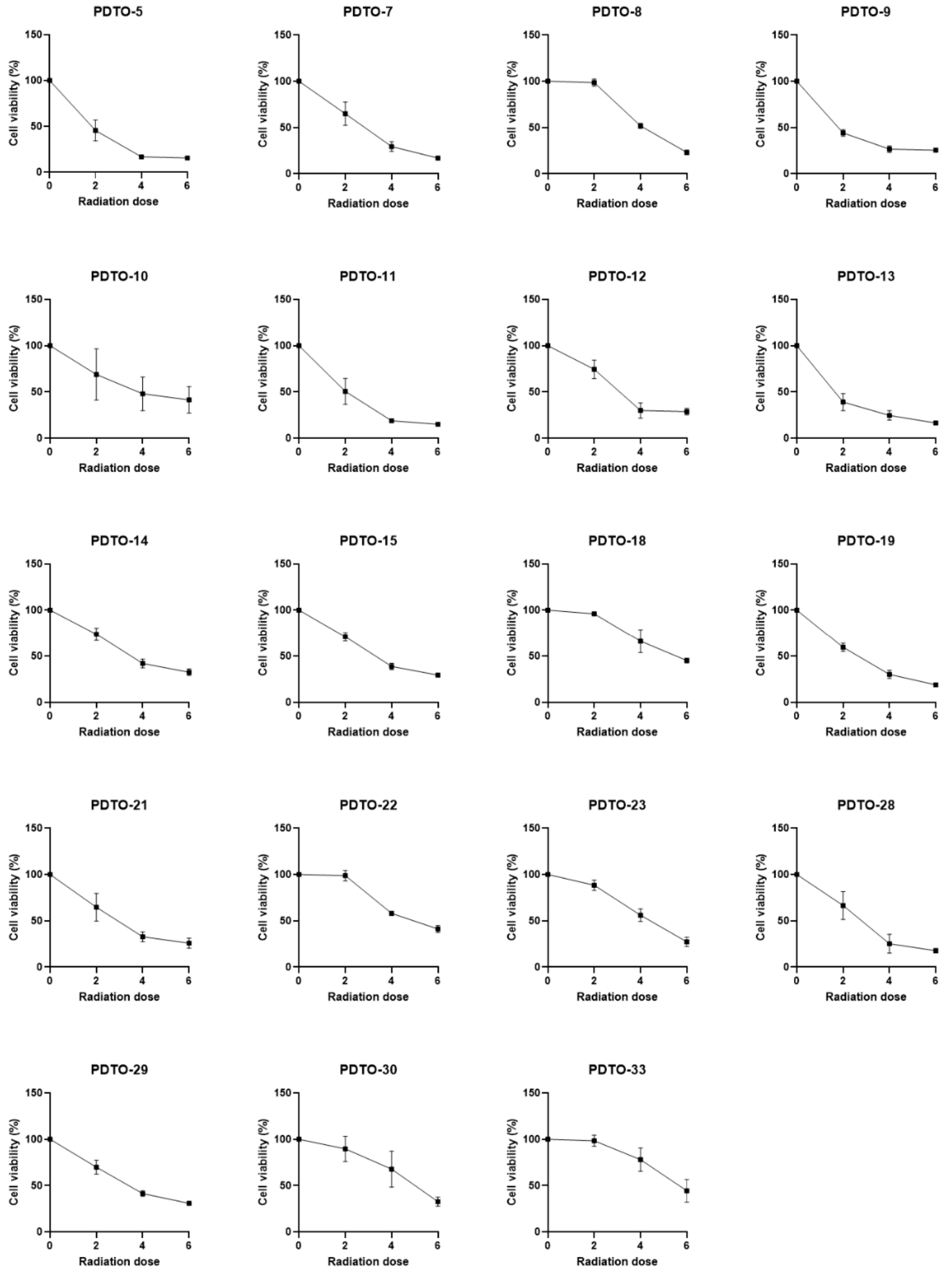

Figure S5. Dose-response of cell viability in 19 PDTOs (n = 6, independent experiments for each PDTO) is shown at 0 Gy, 2 Gy, 4 Gy, and 6 Gy. Data are presented as mean ± standard deviation.
PDTO, patient-derived tumour organoid.

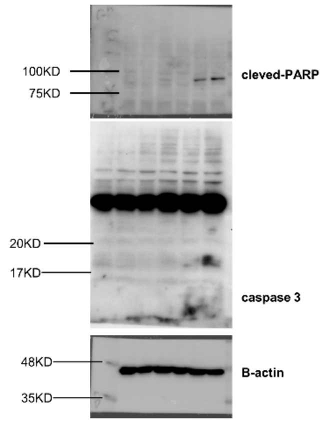

Figure S6. Whole blot showing all the bands with molecular weight marker.

Table S1. List of chemical and reagents used for studies.

Chemical and Reagents	Source	Catalog number	Final concentration.
Matrigel	Corning	356231	
Advanced DMEM/F12	Gibco	12634-010	
HEPES	Gibco	15630-080	1x
Gluta max	Gibco	35050-06	1x
Penicillin-Streptomycin	Gibco	15140-122	1x
B27	Gibco	17504-044	1x
N-acetyl cysteine	United States Pharmacopeia	1009005	1.25mM
human epidermal growth factor	BioVision	4022-100	50ng/ml
human Noggin	Peprotech	120-10c	50ng/ml
gastrin	Sigma-Aldrich	G9145	10nM
A83-01	BioVision	1989-1	500nM
primocin	InvivoGen	ant-pm-1	100ng/ml
Y-27632	BioVision	1784-5	10uM

Table S2. List of antibodies used for studies.

Antibodies	Source	Catalog number	Antibody dilution
anti-Ki-67	Abcam	ab16667	1:200
anti-Muc2	Abcam	ab90007	1:100
anti-E-cadherin	BD Transduction Laboratories	610181	1:200
anti-VL1	Santa Cruz Biotechnology	SC-28283	1:100
anti-ChgA	ImmunoStar	20086	1:100
anti-CDX2	Sigma-Aldrich	235R-16	1:200
anti-CK20	Sigma-Aldrich	320M-16	1:500
anti-CK19	Abcam	ab15463	1:400
anti-cleaved PARP	Cell Signaling Technology	5625	1:1000
anti-caspase 3	Cell Signaling Technology	9662	1:1000
β-actin	Sigma-Aldrich	A5441	1:4000
goat anti-rabbit-Alexa594	Thermo Fisher Scientific	A11012	1:200
goat anti-mouse-Alexa488	Thermo Fisher Scientific	A11001	1:200

Table S3. Results of ROC about two extreme categories. PPV; positive predictive value. NPV; negative predictive value.

		AUC (95% CI)	Sensitivity	Specificity	PPV	NPV
**TRG 0 or not**	D0	0.753 (0.644–0.863)	78.3%	69.2%	52.9%	87.8%
	Survival fraction model	0.897 (0.83–0.965)	95.0%	78.6%	61.3%	97.8%
	Cell viabiiity model	0.631 (0.525–0.737)	89.7%	39.3%	33.8%	91.7%
**TRG 3 or not**	D0	0.966 (0.926–1)	100.0%	93.7%	75.0%	100.0%
	Survival fraction model	0.974 (0.941–1)	100.0%	93.8%	75.0%	100.0%
	Cell viabiiity model	0.898 (0.827–0.968)	77.8%	92.6%	66.7%	100.0%
